# Approximating the uncertainty of deep learning reconstruction predictions in single-pixel imaging

**DOI:** 10.1038/s44172-023-00103-1

**Published:** 2023-08-01

**Authors:** Ruibo Shang, Mikaela A. O’Brien, Fei Wang, Guohai Situ, Geoffrey P. Luke

**Affiliations:** 1 Thayer School of Engineering, Dartmouth College, Hanover, NH 03755 USA; 2 Department of Bioengineering, University of Washington, Seattle, WA 98195 USA; 3 Shanghai Institute of Optics and Fine Mechanics, Chinese Academy of Sciences, Shanghai, 201800 China; 4 Center of Materials Science and Optoelectronics Engineering, University of Chinese Academy of Sciences, Beijing, 100049 China; 5 Hangzhou Institute for Advanced Study, University of Chinese Academy of Sciences, Hangzhou, 310024 China

**Keywords:** Electrical and electronic engineering, Computational science, Imaging and sensing

## Abstract

Single-pixel imaging (SPI) has the advantages of high-speed acquisition over a broad wavelength range and system compactness. Deep learning (DL) is a powerful tool that can achieve higher image quality than conventional reconstruction approaches. Here, we propose a Bayesian convolutional neural network (BCNN) to approximate the uncertainty of the DL predictions in SPI. Each pixel in the predicted image represents a probability distribution rather than an image intensity value, indicating the uncertainty of the prediction. We show that the BCNN uncertainty predictions are correlated to the reconstruction errors. When the BCNN is trained and used in practical applications where the ground truths are unknown, the level of the predicted uncertainty can help to determine whether system, data, or network adjustments are needed. Overall, the proposed BCNN can provide a reliable tool to indicate the confidence levels of DL predictions as well as the quality of the model and dataset for many applications of SPI.

## Abstract (web summary)

Ruibo Shang and coworkers introduce a Bayesian convolutional neural network (BCNN) to estimate the uncertainty of deep learning predictions in single-pixel imaging. The approach is a reliable tool where the confidence of image prediction needs to be approximated, such as simulated training data and hardware adjustments.

## Introduction

Single-pixel imaging (SPI)^1–3^ is a novel imaging technique which uses a single-element photodetector to record the image information instead of using pixel array image sensors. The object is sequentially illuminated by a set of specially designed patterns and the total intensity light for each pattern illumination is collected as a single-pixel value by the photodetector^1^. Finally, computational algorithms are applied to reconstruct the object with the sequential intensity collections and the illumination patterns. SPI has many advantages including high speed, broad bandwidth and compact imaging^4^. It also has many applications including remote sensing^5^, holography^6,7^, optical encryption^8,9^ and tomography^10^.

One of the most common reconstruction methods in SPI is sparsity-based optimization which seeks to reconstruct images from incomplete measurements^11,12^ by incorporating the knowledge that most natural images are sparse when the image is transformed into a specific domain. However, the primary drawback is that it is time consuming because of its iterative nature. An image reconstruction task can take up to hours to compute if the scale of the model or scope of the problem is large. Therefore, real-time imaging is infeasible for applications that require pipelined data acquisition and image reconstruction^13^. Besides, the optimal algorithm-specific parameters (i.e., the regularization parameter) generally need to be heuristically determined^13^.

Deep learning (DL)^14,15^ is an emerging and powerful computational imaging tool dramatically improving the state-of-the-art in image reconstruction compared with conventional reconstruction algorithms^16–22^. It relies on large amounts of training data to automatically learn tasks by finding the optimal weights in each layer of a neural network^15^. This is in contrast to iteratively optimizing the image with a specific model in sparsity-based optimization approaches. Therefore, DL is a promising alternative to augment or replace iterative algorithms in sparsity-based optimization^13^. Researchers have applied DL approaches in SPI to improve the quality of the reconstructed images compared with conventional approaches^13,23–27^. For instance, a DL approach was proposed to predict the image in SPI with an initial guess of the image from conventional approaches as the input to the DL network to further improve the image quality at high compression ratios and noise levels^24^. End-to-end DL approaches^13,26^ were proposed to predict the image in SPI directly from the raw measurement data without the knowledge of the imaging model and therefore no pre-processing of the raw measurement data is needed.

Generally, the accuracy of the DL predictions in SPI can be quantified by comparing with the ground-truth images^13^ (e.g., calculating mean absolute error (MAE), root mean squared error and structural similarity index (SSIM)^28^). However, one outstanding challenge is that the ground truth is usually unknown during the prediction stage in many practical applications. Therefore, the accuracy of the DL prediction of a particular image cannot be estimated.

Bayesian convolutional neural networks (BCNNs) have been shown to be an effective approach to approximate the uncertainty with applications including image segmentation^29^, phase imaging^19^, optical metrology^30^ and image classification^31^. BCNN works on the principle that each pixel in the output image represents the parameter of a probability distribution (e.g., Laplacian or Gaussian distribution), rather than a single intensity value^32^. Then, the uncertainty can be quantified by Monte Carlo dropout^33^ or Deep Ensembles^34^, for example. BCNNs have many advantages over conventional convolutional neural networks (CNNs). One outstanding advantage is that when the prediction of the image fails in a practical application (i.e., the ground truth is unknown), the BCNN is able to provide an alert on predicted images with high uncertainty. With the alert, one could consequently make adjustments for better performance.

In this paper, we propose to use a BCNN in SPI to simultaneously predict the image and the pixel-wise uncertainty to quantify the accuracy of the predicted image. We show the BCNN predictions of the image and uncertainty in both simulated and experimental SPI with analysis in details. Overall, these results show that uncertainty approximation can be used to reliably interpret the result of a compressed computational imaging problem.

## Results

### The BCNN predictions in the simulated SPI trained with the MNIST database

**Fig. 1:**
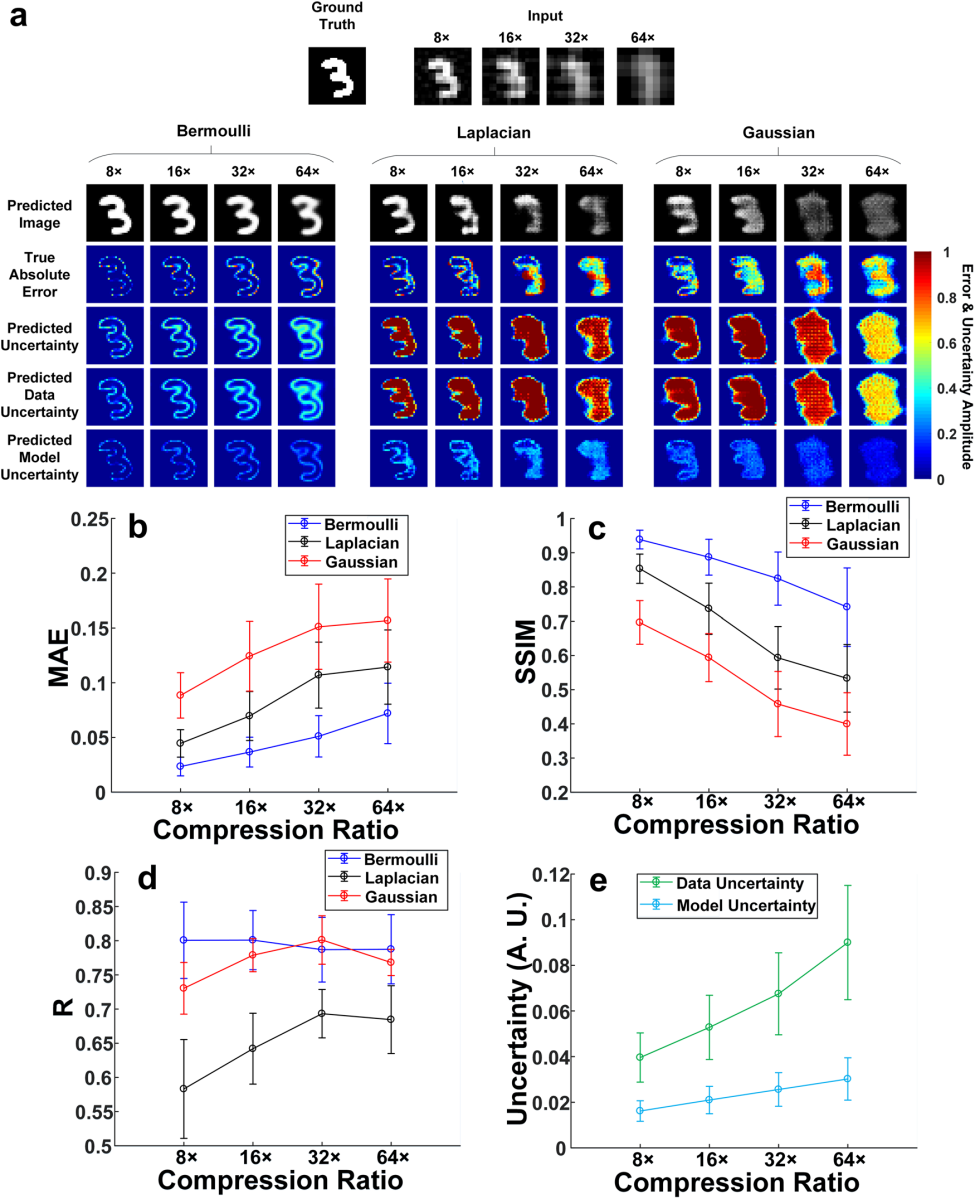
The BCNN predictions in the simulated SPI trained with the MNIST database. **a** A representative ground-truth image in the testing dataset, input images to the BCNN calculated from the LSQR-approximated inverse model matrix and the BCNN predictions with Bernoulli-distributed, Laplacian-distributed and Gaussian-distributed likelihood functions at the 8×, 16×, 32× and 64× compression ratios. **b** The MAEs of the predicted images in BCNN with the three likelihood functions at the four compression ratios. **c** The SSIMs of the predicted images in BCNN with the three likelihood functions at the four compression ratios. **d** The correlation coefficient, R, between the predicted uncertainty and the absolute error of each pixel the predicted images reconstructed with the three likelihood functions at the four compression ratios. **e** Averaged pixel values of the predicted data and model uncertainties in the testing dataset with the Bernoulli-distributed likelihood function at the four compression ratios. The error bars represent the standard deviation of the corresponding parameters from 100 testing images.

Figure 1a shows a representative ground-truth image in the testing dataset, input images to the network calculated from the LSQR-approximated^35^ inverse model matrix and BCNN predictions (with all three likelihood functions) at 8×, 16×, 32× and 64× compression ratios. To quantitatively compare the BCNN predictions with the three likelihood functions, the mean and standard deviation of the MAE and SSIM for all the predicted images, and the correlation coefficient (*R*) between the true absolute error (difference between the ground-truth image and the predicted image) and the predicted uncertainty in the testing dataset at each compression ratio were calculated following Eq. 1 and shown in Fig. 1b–d.

1 $$R=\frac{1}{N-1}\mathop{\sum }\limits_{i=1}^{N}\left(\frac{{A}_{i}-{\mu }_{A}}{{\sigma }_{A}}\right)\left(\frac{{B}_{i}-{\mu }_{B}}{{\sigma }_{B}}\right)$$

where *A* is the true absolute error, *B* is the predicted uncertainty, ${\mu }_{A}$ is the mean of *A*, ${\sigma }_{A}$ is the standard deviation of *A*, ${\mu }_{B}$ is the mean of *B*, ${\sigma }_{B}$ is the standard deviation of *B*, $i$ is the pixel number and *N* is the total number of pixels.

Both the qualitative and quantitative results show that the accuracy of the predicted images from the three likelihood functions decreases as the compression ratio increases. This is verified with an increase of the true absolute error in Fig. 1a, an increase of the MAE in Fig. 1b and a decrease of the SSIM in Fig. 1c. This is reasonable since higher compression ratio means higher model ill-posedness which results in solving a more difficult imaging inverse problem^13^. However, the predicted images in BCNN with the Bernoulli-distributed likelihood function are more accurate than those with the Laplacian-distributed and Gaussian-distributed likelihood functions, especially at higher compression ratios. In terms of the predicted uncertainties, the BCNN with the Bernoulli-distributed likelihood function still performs better than the ones with Laplacian-distributed and Gaussian-distributed likelihood functions. In the BCNN with the Bernoulli-distributed likelihood function, the predicted uncertainties generally match well with the true absolute error. The regions of the predicted image from BCNN with larger errors are generally marked with higher uncertainty values in the predicted uncertainty. It can be observed from the true absolute error and predicted uncertainty that most of the higher inaccuracies come from the edges of the image features. However, the predicted uncertainties in the BCNN with Laplacian-distributed and Gaussian-distributed likelihood functions do not match well with the true absolute error. For instance, as shown in Fig. 1a at the 8× compression ratio, higher true absolute errors in the predicted images of BCNN with the two likelihood functions mostly come from the edges of the image features while the predicted uncertainties indicate higher uncertainties not only on the edges but also within the feature regions. The improved performance of the uncertainty predictions with the Bernoulli-distributed likelihood function can also be quantitatively seen in Fig. 1d with a generally higher correlation coefficient between the predicted uncertainty and the true absolute error.

We also explored the effect of noise on both the image and uncertainty predictions in BCNN (Supplementary Note 1). The results show that the performance of BCNN decreases as the signal-to-noise ratio (SNR) decreases from 25 dB to 0 dB. However, the fidelity of the corresponding result is still at a high level even if the data SNR is as low as 0 dB, suggesting good robustness to noise.

In summary, for the MNIST database^36^, the BCNN with the Bernoulli-distributed likelihood function performs the best among the BCNNs with three distribution likelihood functions. The BCNNs with the Laplacian-distributed and Gaussian-distributed likelihood functions are not suitable for the MNIST database. This is reasonable since the modified images in the MNIST database are binary, which fits with the Bernoulli distribution. In addition, it is observed that the data uncertainty is dominant over the model uncertainty. This effect becomes more pronounced at higher compression ratios. This can be shown quantitatively by the averaged pixel values in the predicted data and model uncertainties in the testing dataset with the Bernoulli-distributed likelihood function in Fig. 1e. We hypothesize that this comes from the compressed nature of the measurement data in the training set of the MNIST database.

### Effect of the physics-prior based preprocessor and uncertainty estimation on network performance

For the BCNN predictions shown in Fig. 1, a pre-processing step was used to convert the inputs of the neural network from measurement domain into image domain. In this section, we sought to explore the effect of the physics-prior based preprocessor to the BCNN performance. We also compared the performance of BCNN with the conventional CNN, which does not have the uncertainty prediction. We denote BCNN as the BCNN with the physics-prior based preprocessor, End-To-End BCNN as the BCNN with the one-dimensional (1D) raw measurement data as the network input, CNN as the CNN with the physics-prior based preprocessor but without the uncertainty prediction function, and End-To-End CNN as the CNN without either the physics-prior based preprocessor or the uncertainty prediction function. To solve the dimension mismatch between the 1D raw measurement data and the two-dimensional image, a fully-connected layer (together with reshape and permute layers) was added in between the input layer and the first convolutional layer of the BCNN to generate End-To-End BCNN. CNN and End-To-End CNN have the same network structures as BCNN and End-To-End BCNN respectively, except that there is no uncertainty prediction incorporated in the loss function. The Bernoulli-distributed likelihood function was used in BCNN and End-To-End BCNN. The training and validation curves are shown in Supplementary Fig. 3.

**Fig. 2:**
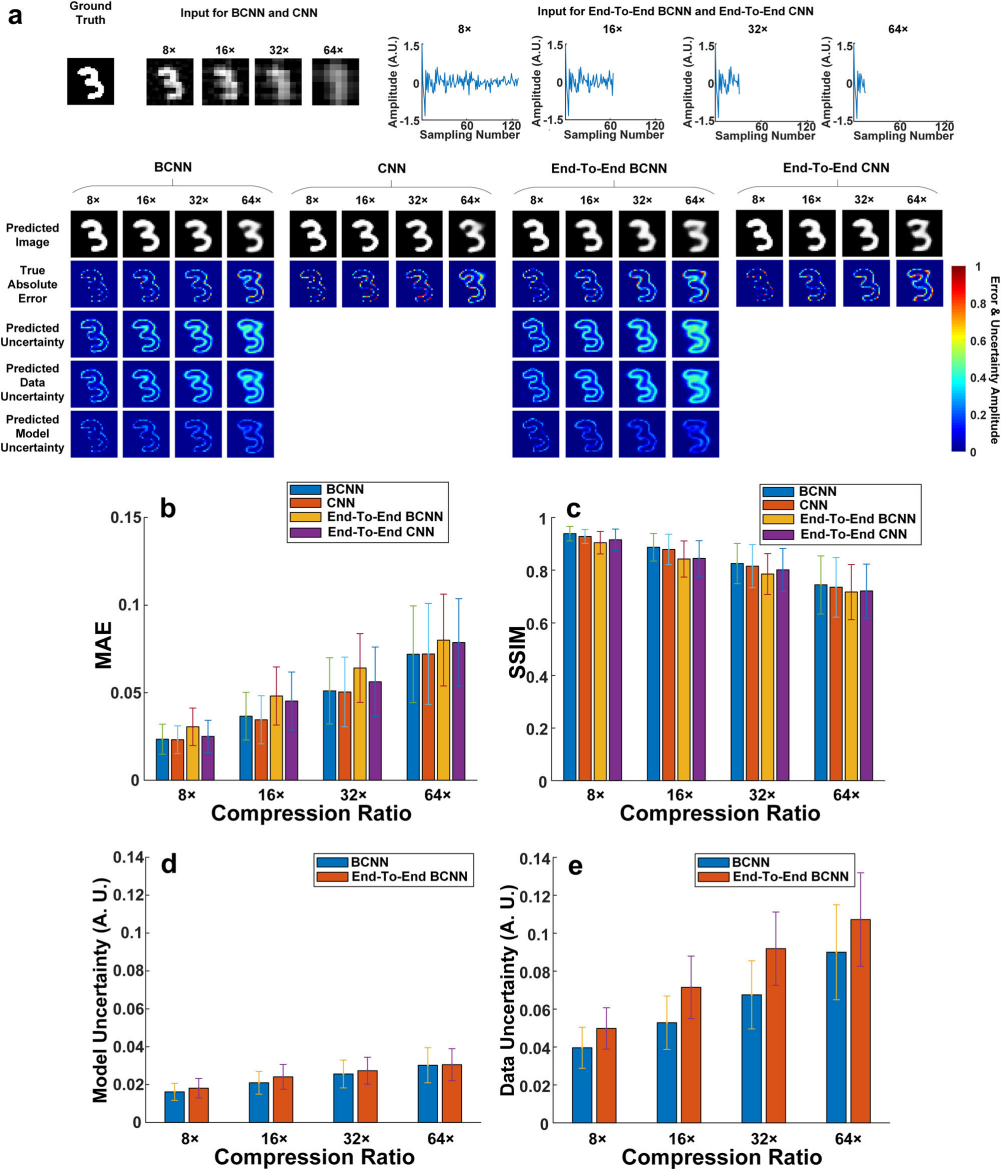
Comparisons among BCNN, CNN, End-To-End BCNN and CNN in the simulated SPI trained with the MNIST database. **a** A representative ground-truth image in the testing dataset, input images to the BCNN and CNN calculated from the LSQR-approximated inverse model matrix, 1D raw measurement data as the input to End-To-End BCNN and End-To-End CNN, and the predictions from BCNN, CNN, End-To-End BCNN and End-To-End CNN at the 8×, 16×, 32× and 64× compression ratios. **b** The MAEs of the predicted images in BCNN, CNN, End-To-End BCNN and End-To-End CNN at the four compression ratios. **c** The SSIMs of the predicted images in BCNN, CNN, End-To-End BCNN and End-To-End CNN at the four compression ratios. **d** Averaged pixel values of the predicted model uncertainties in BCNN and End-To-End BCNN in the testing dataset at the four compression ratios. **e** Averaged pixel values of the predicted data uncertainties in BCNN and End-To-End BCNN in the testing dataset at the four compression ratios. The error bars represent the standard deviation of the corresponding parameters from 100 testing images.

Figure 2a shows the ground-truth image, the input images for BCNN and CNN, the 1D raw measurement data as the input to End-To-End BCNN and End-To-End CNN, and predictions from BCNN, CNN, End-To-End BCNN and End-To-End CNN at 8×, 16×, 32× and 64× compression ratios. To quantitatively compare BCNN, CNN, End-To-End BCNN and End-To-End CNN, the mean and standard deviation of the MAE and SSIM for all the predicted images were calculated and shown in Fig. 2b, c. Figure 2d, e show the averaged pixel values of the predicted model and data uncertainties in BCNN and End-To-End BCNN in the testing dataset at the four compression ratios. The results show that BCNN and CNN have comparable performance on image predictions in terms of MAE and SSIM (Fig. 2b, c), which means that the extra uncertainty predictions in BCNN do not affect its image predictions compared to the conventional CNN. The results also show that BCNN and CNN have better performance in image predictions than End-To-End BCNN and End-To-End CNN in terms of MAE and SSIM. The reason for this outperformance is that BCNN and CNN incorporate physics priors to obtain the initial-guess images as the network inputs to reduce the uncertainty from the data, thus improving the image predictions. This can also be verified in Fig. 2e where BCNN has lower data uncertainties than End-To-End BCNN at all the four compression ratios. Besides, BCNN and End-To-End BCNN have roughly the same model uncertainties at all the four compression ratios since they have similar network structures.

### The BCNN predictions in the simulated SPI trained with the STL-10 database

**Fig. 3:**
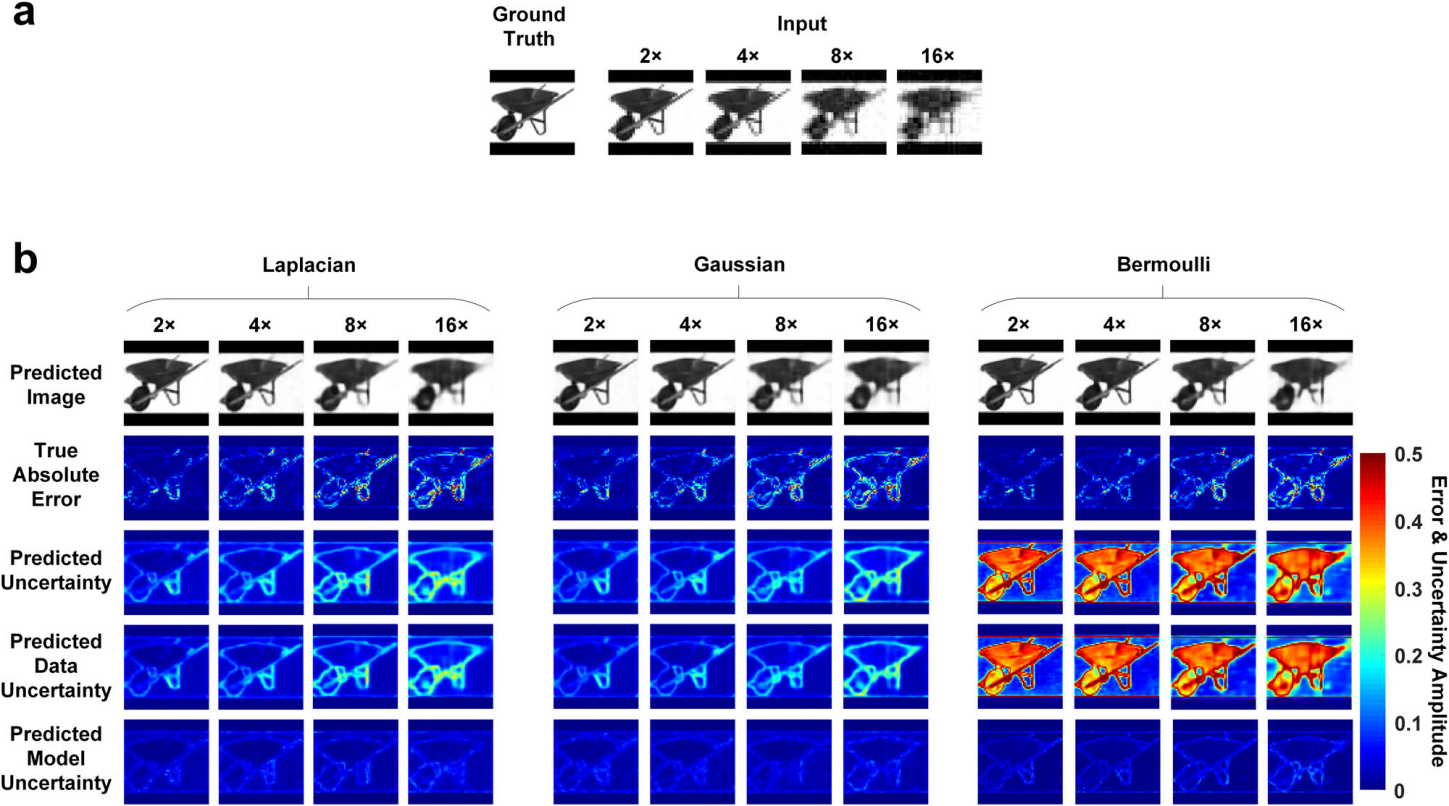
The results of BCNN with Laplacian-distributed, Gaussian-distributed and Bernoulli-distributed likelihood functions in simulated SPI with STL-10 dataset at 2×, 4×, 8× and 16× compression ratios. **a** A representative ground-truth image in the testing dataset, input images to the BCNN calculated from the LSQR-approximated inverse model matrix at the 2×, 4×, 8× and 16× compression ratios. **b** BCNN predictions with Laplacian-distributed, Gaussian-distributed and Bernoulli-distributed likelihood functions at the 2×, 4×, 8× and 16× compression ratios.

In this section, we explore the BCNN performances with the three likelihood functions in the simulated SPI with a more challenging task where the STL-10 database^37^ with more complexed image features is used for training and predictions. Figure 3a shows a representative ground-truth image in the testing dataset, input images to the network calculated from the LSQR-approximated inverse model matrix and Fig. 3b shows BCNN predictions (with all three likelihood functions) at 2×, 4×, 8× and 16× compression ratios. The mean and standard deviation of the MAE and SSIM for all the predicted images, and the correlation coefficient between the true absolute error and the predicted uncertainty in the testing dataset with the three likelihood functions at each compression ratio were calculated and shown in Supplementary Table 1.

The results in Fig. 3 and Supplementary Table 1 show that the accuracy of the predicted images from the three likelihood functions decreases as the compression ratio increases, which is reasonable since higher compression ratio means higher model ill-posedness which results in solving a more difficult imaging inverse problem^13^. Besides, the prediction of the images in BCNNs with the three likelihood functions performs close to each other as shown qualitatively in the predicted-image rows in Fig. 3b and quantitatively in terms of MAE and SSIM in Supplementary Table 1. The predicted uncertainties in BCNNs with the Laplacian-distributed and Gaussian-distributed likelihood functions match well with the true absolute error since the regions where the predicted image from BCNN has larger errors are generally marked with higher uncertainty values in the predicted uncertainty. However, the predicted uncertainties in BCNN with the Bernoulli-distributed likelihood function are much worse as shown qualitatively in Fig. 3b where the low true-absolute-error pixels are marked with higher uncertainty values in the predicted uncertainty instead, and quantitatively in Supplementary Table 1 where the correlation coefficient R between the true absolute error and the predicted uncertainty from the BCNN with the Bernoulli-distributed likelihood function are much lower than those with the Laplacian-distributed and Gaussian-distributed likelihood function. This is reasonable since the loss function for the Bernoulli distribution in Eq. 16 also minimizes the error between the mean of the pixel distribution and the ground truth. Therefore, the predictions of the images perform close to those using the Laplacian-distributed and Gaussian-distributed likelihood function. The uncertainty prediction, however, is dependent on both the predicted mean and the predicted standard deviation. In the case of the Bernoulli distribution and STL-10 dataset, the predicted standard deviation denotes how far the pixel value is from 1 or 0 since it expects a binary image, while the images in the STL-10 database are in gray scale. In this case, the data uncertainty and the overall uncertainty calculated from Eq. 21 will be wrong. The model uncertainty which is the variance of the predicted mean with Monte Carlo Dropout, however, is reasonable since the predicted mean is correct. Therefore, it indicates that when using BCNN to make predictions in SPI with the STL-10 database, the Laplacian-distributed and Gaussian-distributed likelihood functions can be used while the Bernoulli-distributed likelihood function is not suitable. It is also observed that the predicted uncertainty and the true absolute error from the Laplacian-distributed and Gaussian-distributed likelihood functions are only modestly correlated. The modest correlation comes from the fact that not all areas of higher uncertainty necessarily have high error. They merely point out pixels where high errors are likely to occur. A perfect correlation would indicate that perfect reconstruction is possible. Besides, similar to the observations in the other simulations, it can still be observed from the true absolute error and predicted uncertainty that most of the higher inaccuracies come from the edges of the image features, and that the data uncertainty is dominant over the model uncertainty.

We also quantitatively compared BCNN, CNN, End-To-End BCNN and End-To-End CNN with the STL-10 database in the simulated SPI at the 4× compression ratio. The Laplacian-distributed likelihood function was used in BCNN and End-To-End BCNN. The results are shown in Supplementary Fig. 4. Similar to the comparisons of the four neural networks with the MNIST database, BCNN and CNN have similar performance on image predictions in terms of MAE and SSIM, which means that the extra uncertainty predictions in BCNN do not affect its image predictions compared to the conventional CNN. The results also show that BCNN and CNN have better overall performance in image predictions than End-To-End BCNN and End-To-End CNN in terms of MAE and SSIM.

Thus far, the BCNN was applied separately to either the MNIST and STL-10 database. We also explored a different training strategy where the BCNN was trained with a mixture of the MNIST and STL-10 databases (Hybrid Training) with either Laplacian-distributed or Bernoulli-distributed likelihood functions. We quantitatively compared its performance with the one trained on the two databases separately (Separate Training) in Supplementary Note 2. The results show that Separate Training has better overall performance in image and uncertainty predictions than Hybrid Training.

### Experimental results

**Fig. 4:**
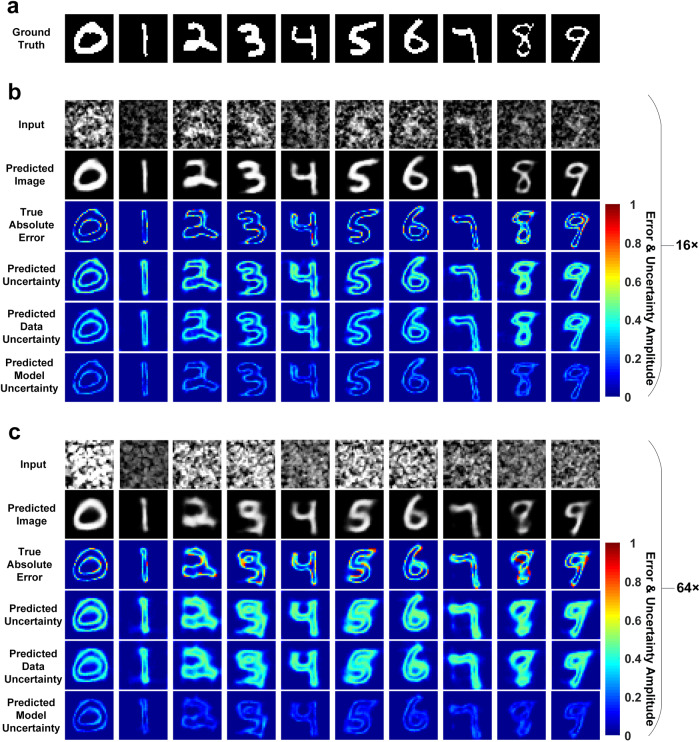
Experiment results with BCNN in SPI with the MNIST dataset at 16× and 64× compression ratios. **a** Ten representative ground-truth images from the MNIST testing dataset. **b** Input images and predictions of BCNN in SPI at 16× compression ratio. **c** Input images and predictions of BCNN in SPI at 64× compression ratio.

**Fig. 5:**
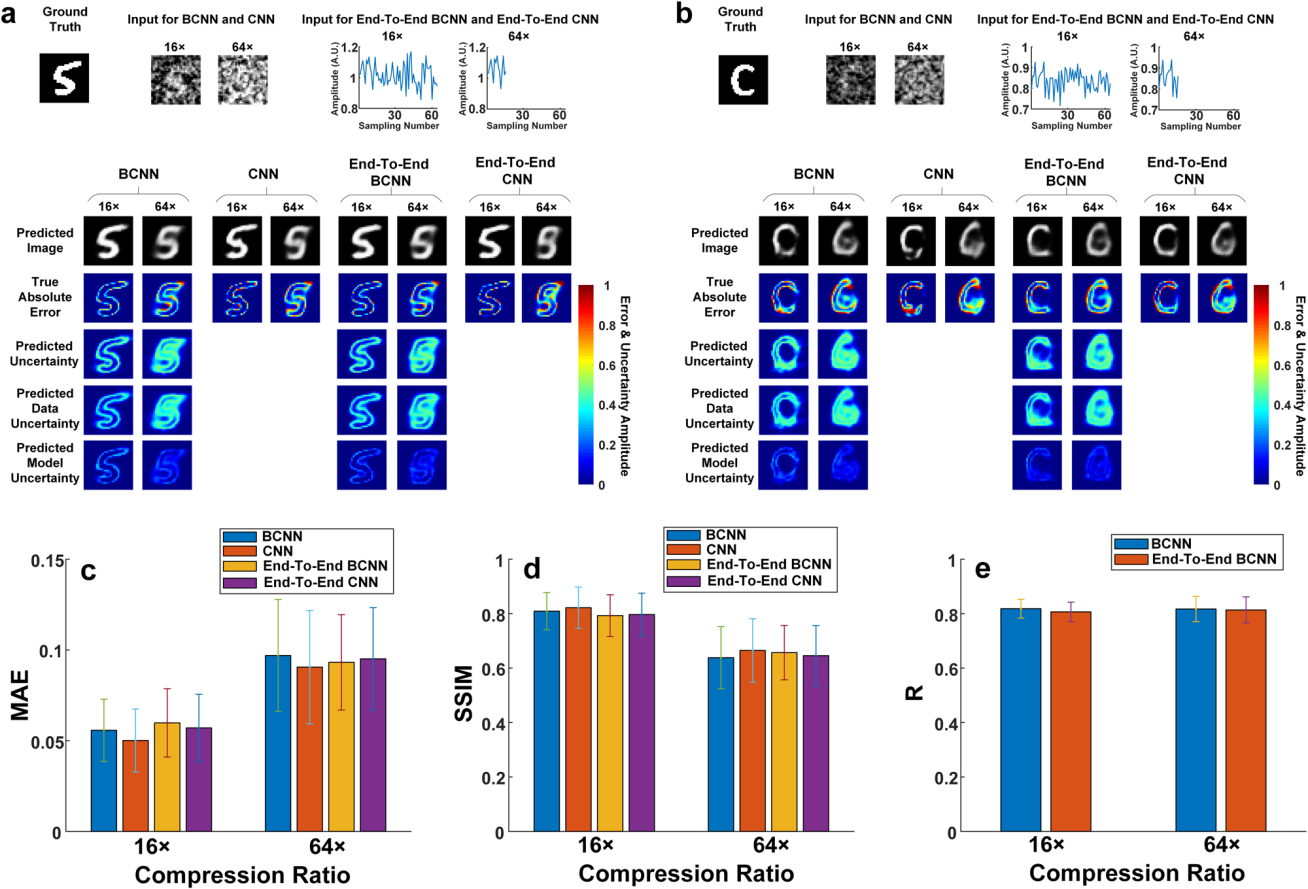
Comparisons among BCNN, CNN, End-To-End BCNN and CNN in the experimental SPI trained with the MNIST database. **a** A representative ground-truth image in the testing dataset, input images to the BCNN and CNN calculated from the LSQR-approximated inverse model matrix, 1D raw measurement data as the input to End-To-End BCNN and End-To-End CNN, and the predictions from BCNN, CNN, End-To-End BCNN and End-To-End CNN at the 16× and 64× compression ratios. **b** A ground-truth image out of the testing dataset in the MNIST database, input images to the BCNN and CNN calculated from the LSQR-approximated inverse model matrix, 1D raw measurement data as the input to End-To-End BCNN and End-To-End CNN, and the predictions from BCNN, CNN, End-To-End BCNN and End-To-End CNN at the 16× and 64× compression ratios. **c** The MAEs of the predicted images in BCNN, CNN, End-To-End BCNN and End-To-End CNN at the two compression ratios. **d** The SSIMs of the predicted images in BCNN, CNN, End-To-End BCNN and End-To-End CNN at the two compression ratios. **e** The correlation coefficient, R, between the predicted uncertainty and the true absolute error of each pixel the predicted images reconstructed in BCNN and End-To-End BCNN at the two compression ratios. The error bars represent the standard deviation of the corresponding parameters from 100 testing images.

Figure 4a shows representative ground-truth images from the testing dataset. Input images and the predictions of BCNN at 16× and 64× compression ratios are shown in Fig. 4b, c. The BCNN provides reasonably good predicted images in SPI at both 16× and 64× compression ratios. However, the images at 64× are in poorer quality than those at 16×. This can be visualized from the true absolute errors in Fig. 4b, c. Quantitative results of BCNN predictions are shown in Fig. 5c–e. In terms of the predicted images, the performance of the BCNN decreases as the compression ratio increases from 16× to 64×. However, it still remains at a good level with an MAE lower than 0.1 and an SSIM higher than 0.6. The performance of the BCNN in terms of the predicted uncertainty remains at almost the same level at the two compression ratios, showing its great robustness. Visually, the predicted uncertainty generally matches well with the true absolute error in Fig. 4b, c. The regions where the predicted image from BCNN has larger errors are generally marked with higher uncertainty values in the predicted uncertainty. It can still be observed from the true absolute error and predicted uncertainty that most of the higher inaccuracies come from the edges of the image features. Again, the data uncertainty is dominant over the model uncertainty due to the compressed nature and noise in the training dataset.

We also quantitatively compared BCNN, CNN, End-To-End BCNN and End-To-End CNN in this experimental SPI with the MNIST database. Figure 5a shows a representative ground-truth image, the input images for BCNN and CNN, the 1D raw measurement data as the input to End-To-End BCNN and End-To-End CNN, and predictions from BCNN, CNN, End-To-End BCNN and End-To-End CNN at 16× and 64× compression ratios. Figure 5b shows a representative ground-truth image out of the MNIST database, the input images for BCNN and CNN, the 1D raw measurement data as the input to End-To-End BCNN and End-To-End CNN, and predictions from BCNN, CNN, End-To-End BCNN and End-To-End CNN at 16× and 64× compression ratios. It shows that BCNN has good generalization performance in the experimental SPI. To quantitatively compare BCNN, CNN, End-To-End BCNN and End-To-End CNN, the mean and standard deviation of the MAE and SSIM for all the predicted images were calculated and are shown in Fig. 5c, d. Figure 5e shows the mean and standard deviation of the correlation coefficient R in BCNN and End-To-End BCNN for all the predicted images. The training and validation curves are shown in Supplementary Fig. 5. The results show that CNN has only slightly better performance than the BCNN (Fig. 5c, d), which means that the extra uncertainty predictions in BCNN do not appreciably affect their image predictions compared to the conventional CNN. The results also show that BCNN and CNN have similar performance in image predictions compared to End-To-End BCNN and End-To-End CNN in terms of MAE and SSIM, which is slightly different from the corresponding conclusion in the simulated case. The reason for the difference is that random grayscale patterns were used in the experiments instead of Russian-Doll (RD) Hadamard patterns used in the simulation, leading to a more ill-posed inverse problem. Therefore, even though BCNN and CNN incorporate physics priors to obtain the initial-guess images as the network inputs, the data uncertainty is not reduced, thus not improving the image predictions. Besides, BCNN and End-To-End BCNN have roughly the same correlation coefficient R at both compression ratios as shown in Fig. 5e, indicating that they have similar performance in uncertainty predictions.

## Discussion

The BCNN is proposed for uncertainty approximation in SPI with Bernoulli-distributed, Laplacian-distributed or Gaussian-distributed likelihood functions with the MNIST and STL-10 databases. First, the BCNNs with the three distribution likelihood functions were compared in simulated SPI with the MNIST database at varying compression ratios and the Bernoulli-distributed likelihood function was proved to be the most appropriate among the three functions. Second, the robustness of BCNN to noise from the measurement data was studied with the Bernoulli-distributed likelihood function and the MNIST database (Supplementary Note 1). Third, the three likelihood functions were compared in BCNN in simulated SPI with STL-10 dataset and the Laplacian-distributed and Gaussian-distributed likelihood functions were shown to be equivalent and both better than the Bernoulli-distributed likelihood function in this application. Fourth, different training strategies were compared in Supplementary Note 2. Fifth, in experiments, the BCNN with Bernoulli-distributed likelihood functions was used and verified in experimental SPI with the MNIST dataset at 16× and 64× compression ratios. In all the simulations and experiments, the performances of BCNN, CNN, End-To-End BCNN and End-To-End CNN were quantitatively evaluated to study the effect of the physics-prior based preprocessor and the uncertainty estimation on network performance.

BCNNs have advantages over conventional CNNs. As shown in Figs. 1–5, BCNNs not only predict the image as conventional CNNs do but also provide a reliability assessment to indicate the pixel-wise uncertainties of DL predictions as well as the quality of the model and dataset with model uncertainty and data uncertainty respectively. As the quality of the predicted images decreases, the corresponding uncertainty values increase to indicate this change of the quality. For a specific predicted image, the pixel-wise uncertainty prediction tells the error of each pixel in the predicted image and highlights where the large errors occur in the predicted image. This is especially useful to evaluate the prediction of the neural network when the ground truth is unknown in many practical applications, and the level of the predicted uncertainty can be used to determine whether some adjustments in the imaging system, training data, and/or network architecture are needed.

However, several aspects of this work still need further exploration. First, how to choose the optimal probability-distributed likelihood function in BCNN efficiently is a problem that needs to be explored. In this work, we trained the BCNN with the potential probability-distributed likelihood functions and then compared the results to find the optimal one. However, the drawback is that it is time consuming. Based on our experience, the Bernoulli-distributed likelihood function works well on binary images, and the Laplacian-distributed and Gaussian-distributed likelihood functions work equally well on natural grayscale images. We would like to propose a method to find the optimal distribution before training using the dataset statistics and network architecture. Second, it is observed that the data uncertainty is dominant over the model uncertainty in both simulations and experiments due to the compressed nature and noise in the measurement data in the training dataset. We would like to search for more advanced pre-processing approaches to decrease the uncertainty stemming from the measurement data. Third, we would like to explore ways to use the predicted uncertainty as a feedback to optimize the BCNN structures to further decrease the uncertainty of the results, or to decrease the network complexity without a loss in performance.

In summary, the proposed BCNN enables uncertainty approximation in SPI. It is a reliable tool in diverse applications in SPI where the confidence on the predicted images needs to be approximated.

## Methods

### Mathematical basis of SPI

Here, we focus our discussion on two-dimensional imaging in SPI. We denote the object as ${O}(x,y)$, and the set of patterns used to illuminate the object as ${P}_{m}\,(x,y)$, where *m* = *1,2,…,M* (*M* is the total number of patterns). The 1D signal acquired in SPI can be written as,

2 $${I}_{m}=\int {P}_{m}(x,y)O(x,y){dxdy}$$

When the signal is sampled at discrete pixel locations, Eq. (2) can be written as,

3 $${I}_{m}=\mathop{\sum }\limits_{a=1}^{{Nx}}\mathop{\sum }\limits_{b=1}^{{Ny}}{P}_{m}({x}_{a},{y}_{b})O({x}_{a},{y}_{b})$$

where ${x}_{a}$ and ${y}_{b}$ denote discrete pixel locations. ${N}_{x}$ and ${N}_{y}$ denote the total pixel numbers in $x$ and $y$ dimensions.

Equation (3) represents a linear model, and can be written as matrix multiplication by

4 $$g={{{{{\bf{H}}}}}}f+n$$

where $f$ is the vectorized version of the object $O$ (the dimension of $f$ is ${N}_{x}{N}_{y}\times 1$), $g$ is the raw measurement (the dimension of $g$ is $M\times 1$), $n$ is the noise and ${{{{{\bf{H}}}}}}$ is the forward operator, where the *m*^th^ row of **H** contains the vectorized version of the illumination pattern ${P}_{m}$ (the dimension of ${{{{{\bf{H}}}}}}$ **is** $M\times {N}_{x}{N}_{y}$).

The inverse problem of Eq. (4) is ill-posed due to the compression property of SPI. Therefore, a regularized optimization approach is usually used in SPI to incorporate additional knowledge about the image by adding a regularization term,

5 $$\hat{f}=\mathop{{{{{{\mathrm{arg}}}}}}\,{{{{{\mathrm{min}}}}}}}\limits_{f}\,\left\{\|{{{{{{\bf{H}}}}}}f-g\|}_{2}^{2}+\lambda {{{{{\boldsymbol{\phi}}}}}}(f)\right\}$$

where ${{{{{\boldsymbol{\phi}}}}}}$ is the regularization operator and λ is the regularization parameter. ${\|{{{{{\bf{H}}}}}}f-g\|}_{2}^{2}$ is the fidelity term and ${{{{{\boldsymbol{\phi}}}}}}(f)$ is the regularization term. Common regularization domains include spatial, edge, and wavelet domains. Equation (5) can be solved by iterative optimization approaches or deep learning approaches.

### Bayesian networks for uncertainty approximation

As opposed to conventional convolutional neural networks where the weights are deterministic after training, BCNNs use distributions over the network parameters to replace the deterministic weights in the network^32^. This probabilistic property of BCNN come from the stochastic (random) processes in the network such as dropout^38^, weight initialization^39^ etc. Suppose the training dataset is denoted as $\left(X,Y\right)={\{{x}_{n},{y}_{n}\}}_{n=1}^{N}$ with *X* and *Y* representing the network inputs and ground-truth images, respectively. *N* is the total number of images in the training dataset. To approximate the variability of the prediction y given a specific input ${x}_{{test},t}\,$ in the testing dataset $\left({X}_{{test}},{Y}_{{test}}\right)={\{{x}_{{test},{t}},{y}_{{test},t}\}}_{t=1}^{T}$(*T* is the total number of images in the testing dataset), we use the predictive distribution $p({y|}{x}_{{test},t},X,Y)$ over all possible learned weights (with marginalization)^33^:

6 $$p\left(y|{x}_{{test},t},X,Y\right)=\int p(y{{{{{\rm{|}}}}}}{x}_{{test},t},W)p(W{{{{{\rm{|}}}}}}X,Y){dW}$$

where $p(y{{{{{\rm{|}}}}}}{x}_{{test},t},W)$ denotes the predictive distribution that includes all possible output predictions given the learned weights $W$ and the input ${x}_{{test},t}$ from the testing dataset. It can be understood as data uncertainty^19^. $p(W{{{{{\rm{|}}}}}}X,Y)$ denotes all possible learned weights given the training dataset, which can be understood as model uncertainty^19^.

To model the data uncertainty, we need to define the probability distribution of the BCNN outputs with a specific likelihood function. In this paper, we choose the multivariate Laplacian-distributed, Gaussian-distributed and Bernoulli-distributed likelihood functions to model the data uncertainty.

- We define the multivariate Laplacian-distributed likelihood function as:
  7 $${p}_{{Laplacian}}\left(y|x,W\right)=\mathop{\prod }\limits_{m=1}^{M}{p}_{{Laplacian}}\left({y}^{m}|x,W\right)$$
  8 $${p}_{{Laplacian}}\left({y}^{m}|x,{{{{{\rm{W}}}}}}\right)=\frac{1}{2{\sigma }^{m}}{{\exp }}\left(-\frac{\left|{y}^{m}-{\mu }^{m}\right|}{{\sigma }^{m}}\right)$$
  where *m* denotes the *m*^th^ pixel in the BCNN output image, *M* denotes the total number of pixels in the BCNN output image, and ${\mu }^{m}$ and ${\sigma }^{m}$ denote the mean and standard deviation of the mth pixel in the BCNN output image, respectively.
  By taking logarithm and negative operations on Eq. (7), the loss function ${L}_{{Laplacian}}\left({W|}{x}_{n},{y}_{n}\right)$ for the Laplacian-distributed likelihood function given the training data pair (*x*_*n*_*, y*_*n*_) is:
  9 $${L}_{{Laplacian}}\left(W{{{{{\rm{|}}}}}}{x}_{n},{y}_{n}\right)=\frac{1}{M}\mathop{\sum }\limits_{m=1}^{M}\left[\frac{\left|{y}_{n}^{m}-{\mu }_{n}^{m}\right|}{{\sigma }_{n}^{m}}+{{\log }}(2{\sigma }_{n}^{m})\right]$$
- For multivariate Gaussian-distributed likelihood function, we define:
  10 $${p}_{{Gaussian}}\left(y|x,W\right)=\mathop{\prod }\limits_{m=1}^{M}{p}_{{Gaussian}}\left({y}^{m}|x,W\right)$$
  11 $${p}_{{Gaussian}}\left({y}^{m}|x,{{{{{\rm{W}}}}}}\right)=\frac{1}{\sqrt{2\pi }{\sigma }^{m}}{{\exp }}\left[-\frac{{\left({y}^{m}-{\mu }^{m}\right)}^{2}}{2{({\sigma }^{m})}^{2}}\right]$$
  where the denotations are the same as those in Eqs. (7) and (8).
  By taking logarithm and negative operations on Eq. (10), the loss function ${L}_{{Gaussian}}\left({W|}{x}_{n},{y}_{n}\right)$ for the Gaussian-distributed likelihood function given the training data pair (*x*_*n*_*, y*_*n*_) is:
  12 $${L}_{{Gaussian}}\left(W{{{{{\rm{|}}}}}}{x}_{n},{y}_{n}\right)=\frac{1}{M}\mathop{\sum }\limits_{m=1}^{M}\left[\frac{{({y}_{n}^{m}-{\mu }_{n}^{m})}^{2}}{{2({\sigma }_{n}^{m})}^{2}}+{{\log }}(\sqrt{2\pi }{\sigma }_{n}^{m})\right]$$
- For Bernoulli-distributed likelihood function, we define:

13 $${p}_{{Bernoulli}}\left(y|x,W\right)=\mathop{\prod }\limits_{m=1}^{M}{p}_{{Bernoulli}}\left({y}^{m}|x,W\right)$$

14 $${p}_{{Bernoulli}}\left({y}^{m}=1|x,{{{{{\rm{W}}}}}}\right)={\mu }^{m}$$

15 $${p}_{{Bernoulli}}\left({y}^{m}|x,{{{{{\rm{W}}}}}}\right)={({\mu }^{m})}^{{y}^{m}}{({1-\mu }^{m})}^{{1-y}^{m}}$$

where the denotations are the same as those in Eqs. (7) and (8).

By taking logarithm and negative operations on Eq. (13), the loss function ${L}_{{Bernoulli}}\left({W|}{x}_{n},{y}_{n}\right)$ for the Bernoulli-distributed likelihood function given the training data pair (*x*_*n*_*, y*_*n*_) is:

16 $${L}_{{Bernoulli}}\left(W{{{{{\rm{|}}}}}}{x}_{n},{y}_{n}\right)=\mathop{\sum}\limits_{m=1}^{M}[({y}_{n}^{m}-1){{\log }}(1-{\mu }_{n}^{m})-\,{y}_{n}^{m}{{\log }}({\mu }_{n}^{m})]$$

We would like to learn the weights to maximize Eqs. (7), (10) and (13) in the training dataset, which is equivalent to minimizing the loss functions defined in Eqs. (9), (12) and (16). There are two channels ($\mu$ and $\sigma$) in the BCNN output for Laplacian-distributed and Gaussian-distributed likelihood functions while there is only one channel ($\mu$) in the BCNN output for the Bernoulli-distributed likelihood function.

To measure the model uncertainty, we use the dropout network^33^. A distribution $q(W)$ is learned to approximate $p(W{{{{{\rm{|}}}}}}X,Y)$ (minimizing the Kullback-Leibler divergence between $q(W)$ and $p(W{{{{{\rm{|}}}}}}X,Y)$) by applying a dropout layer before every layer that has learnable weights. During the prediction process, the model uncertainty is approximated by Monte Carlo dropout^33^. With Monte Carlo integration, the predictive distribution $p({y|}{x}_{{test},t},X,Y)$ in Eq. (6) can be approximated as:

17 $$p\left(y|{x}_{{test},t},X,Y\right)\approx \int p(y{{{{{\rm{|}}}}}}{x}_{{test},t},W)q(W){dW}\approx \frac{1}{K}\mathop{\sum }\limits_{k=1}^{K}p(y{{{{{\rm{|}}}}}}{x}_{{test},t},{W}^{k})$$

where *K* is the total number of dropout activations during the prediction process.

Finally, the predicted image can be represented by the predicted mean ${\hat{\mu }}_{{test},t}^{m}$ of the mth pixel for the testing data ${x}_{{test},t}$ (for Laplacian-distributed, Gaussian-distributed and Bernoulli-distributed likelihood functions) is:

18 $${\hat{\mu }}_{{test},t}^{m}={\mathbb{E}}[{y}^{m}{{{{{\rm{|}}}}}}{x}_{{test},t},X,Y]\approx \frac{1}{K}\mathop{\sum}\limits_{k=1}^{K}{\mathbb{E}}[{y}^{m}{{{{{\rm{|}}}}}}{x}_{{test},t},{W}^{k}]\approx \frac{1}{K}\mathop{\sum}\limits_{k=1}^{K}{\hat{\mu }}_{{test},t}^{m,k}$$

where ${\mathbb{E}}$ denotes the expectation and ${\hat{\mu }}_{{test},t}^{m,k}$ denotes the predicted $\mu$ of the *m*^th^ pixel and kth dropout activation for the testing data ${x}_{{test},t}$.

The predicted uncertainty ${\hat{\sigma }}_{{test},t}^{m}$ of the mth pixel for the testing data ${x}_{{test},t}$ for Laplacian-distributed likelihood function is:

19 $${\hat{\sigma}}_{{test},t\left({Laplacian}\right)}^{m} 	= \, \sqrt{{Var}\left({y}^{m}|{x}_{{test},t},X,Y\right)} \\ 	= \, \sqrt{{\mathbb{E}}\left[{Var}\left({y}^{m}|{x}_{{test},t},W,X,Y\right)\right]+{Var}\left({\mathbb{E}}\left[{y}^{m}|{x}_{{test},t},W,X,Y\right]\right)}\\ 	= \, \sqrt{{\mathbb{E}}\left[{Var}\left({y}^{m}|{x}_{{test},t},W\right)\right]+{Var}\left({\mathbb{E}}\left[{y}^{m}|{x}_{{test},t},W\right]\right)}\\ 	 \approx \, \sqrt{\frac{1}{K}\mathop{\sum }\nolimits_{k=1}^{K}2{\left({\hat{\sigma }}_{{test},t}^{m,k}\right)}^{2}+\frac{1}{K}\mathop{\sum }\nolimits_{k=1}^{K}{\left({\hat{\mu }}_{{test},t}^{m,k}-{\hat{\mu }}_{{test},t}^{m}\right)}^{2}} =\sqrt{{\left({\hat{\sigma }}_{{test},t}^{m(D)}\right)}^{2}+{\left({\hat{\sigma }}_{{test},t}^{m(M)}\right)}^{2}}$$

where ${Var}$ denotes pixel-wise variance, ${\hat{\sigma }}_{{test},t}^{m,k}$ denotes the predicted standard deviation of the *m*^th^ pixel and kth dropout activation for the testing data ${x}_{{test},t}$. ${\hat{\sigma }}_{{test},t}^{m(D)}=\sqrt{\frac{1}{K}\mathop{\sum }\nolimits_{k=1}^{K}2{({\hat{\sigma }}_{{test},t}^{m,k})}^{2}}\,$ denotes the data uncertainty and ${\hat{\sigma }}_{{test},t}^{m(M)}=\sqrt{\frac{1}{K}{\sum }_{k=1}^{K}{({\hat{\mu }}_{{test},t}^{m,k}-{\hat{\mu }}_{{test},t}^{m})}^{2}}$ denotes the model uncertainty.

For Gaussian-distributed likelihood function:

20 $${\hat{\sigma }}_{{test},t({Gaussian})}^{m} 	 \approx \sqrt{\frac{1}{K}\mathop{\sum }\nolimits_{k=1}^{K}{\left({\hat{\sigma }}_{{test},t}^{m,k}\right)}^{2}+\frac{1}{K}\mathop{\sum }\nolimits_{k=1}^{K}{\left({\hat{\mu }}_{{test},t}^{m,k}-{\hat{\mu }}_{{test},t}^{m}\right)}^{2}}\\ 	 = \sqrt{{\left({\hat{\sigma }}_{{test},t}^{m(D)}\right)}^{2}+{\left({\hat{\sigma }}_{{test},t}^{m(M)}\right)}^{2}}$$

where the denotations are the same as those in Eq. (19) and the derivation of Eq. (20) is similar to that of Eq. (19).

For Bernoulli-distributed likelihood function:

21 $${\hat{\sigma }}_{{test},t({Bernoulli})}^{m} 	 \approx \sqrt{\frac{1}{K}\mathop{\sum}\nolimits_{k=1}^{K}[{\hat{\mu }}_{{test},t}^{m,k}(1-{\hat{\mu }}_{{test},t}^{m,k})]+\frac{1}{K}\mathop{\sum}\nolimits_{k=1}^{K}{\left({\hat{\mu }}_{{test},t}^{m,k}-{\hat{\mu }}_{{test},t}^{m}\right)}^{2}}\\ 	 = \sqrt{{\left({\hat{\sigma }}_{{test},t}^{m(D)}\right)}^{2}+{\left({\hat{\sigma }}_{{test},t}^{m(M)}\right)}^{2}}$$

where the denotations are the same as those in Eq. (19) and the derivation of Eq. (21) is similar to that of Eq. (19).

We can find from Eqs. (19–21) that the data uncertainty (${\hat{\sigma }}_{{test},t}^{m(D)}$) is approximated by the mean of the predicted variance and the model uncertainty (${\hat{\sigma }}_{{test},t}^{m(M)}$) is approximated by the variance of the predicted mean.

### BCNN structures

**Fig. 6:**
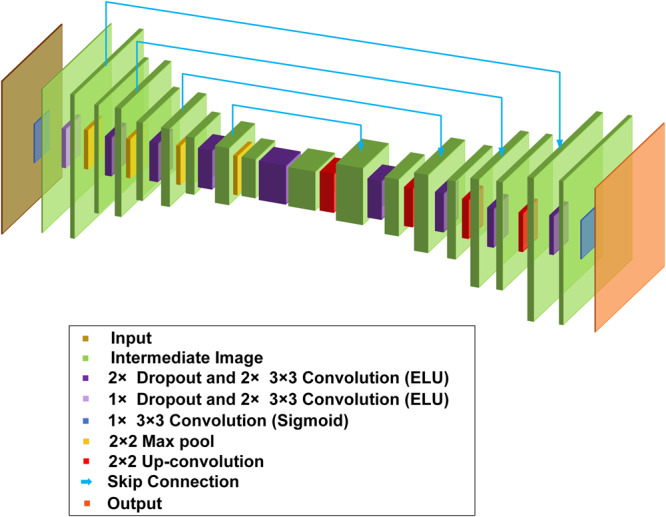
The BCNN structure. The BCNN uses a U-Net architecture with an encoder-decoder structure. Each level of the U-Net includes dropout and convolutional (with ELU or Sigmoid activations) layers. The encoder and decoder are connected through skip connections.

The BCNN structures are shown in Fig. 6. They follow the U-Net architecture^40^, which utilizes an encoder-decoder structure with skip connections to preserve wide-frequency features. This architecture was chosen because of its success in solving image-to-image problems. Dropout layers with a dropout rate of 0.1 were included before each convolution layer of the U-Net in order to prevent overfitting during the training process. L_2_ kernel regularizer and bias regularizer with the regularization factor of $1\times {10}^{-6}$ were included in each convolution layer. The network structure in Fig. 6 is used for Bernoulli-distributed likelihood function. For Laplacian-distributed and Gaussian-distributed likelihood functions, the same architecture is used except that there are two output channels (for $\mu$ and $\sigma$). The loss functions in Eqs. (9), (12) and (16) were used in BCNN for Laplacian-distributed, Gaussian-distributed and Bernoulli-distributed likelihood function, respectively. The BCNN was trained on a NVIDIA Quadro M4000 GPU with an 8GB of memory.

### Data simulation and pre-processing

RD Hadamard^41^ patterns are used as the sampling patterns in the simulated SPI. In RD Hadamard patterns, the measurement order of the Hadamard basis is reordered and optimized according to their significance for general scenes, such that at discretized increments, the complete sampling for different spatial frequencies is obtained^41^.

The MNIST database^36^ was used for training the BCNN with 800 images as the training dataset, 100 images as the validating dataset and another 100 images as the testing dataset. All the images were normalized, converted to binary images and up-sampled from 28 × 28 to 32 × 32 to meet the dimension requirement of the RD Hadamard patterns. The full RD Hadamard basis for a 32 × 32 image has 1024 RD Hadamard patterns each with a size of 32 × 32. Varying compression ratios (varying levels of model ill-posedness) were used here as 8×, 16×, 32× and 64× corresponding to taking the first 1/8, 1/16, 1/32 and 1/64 of the RD Hadamard patterns, respectively. The 1D raw measurement data were acquired by multiplying each individual image with the RD Hadamard patterns at each compression ratio. Therefore, the 1D raw measurement data have a size of 128 × 1, 64 × 1, 32 × 1 and 16 × 1 for the corresponding compression ratios. Finally, white Gaussian noise was added to the 1D measurement data to achieve an SNR of 25 dB. The SNR is defined as:

22 $${SNR}=20\, {{{\log }}}_{10}\frac{{averaged}\,{signal}\,{amplitude}}{{standard}\,{deviation}\,{of\; noise}}$$

The STL-10 natural image database^37^ was used for training the BCNN with 10,000 images as the training dataset, 2000 images as the validating dataset and another 2000 images as the testing dataset. All the images were down-sampled from 96 × 96 to 64 × 64 to meet the dimension requirement of the RD Hadamard patterns. The full RD Hadamard basis for a 64 × 64 image has 4096 RD Hadamard patterns each with a size of 64 × 64. Varying compression ratios (varying levels of model ill-posedness) were used here as 2×, 4×, 8× and 16×. Therefore, the 1D raw measurement data have a size of 2048 × 1, 1024 × 1, 512 × 1 and 256 × 1 for the corresponding compression ratios. Finally, white Gaussian noise was added to the 1D measurement data to achieve an SNR of 25 dB.

In DL, a pre-processing step is usually used to convert the inputs of the neural network from measurement domain into image domain and therefore makes the learning process easier^21,24,42,43^. In this paper, the pre-processing step is a linear operation on the acquired raw SPI data to reconstruct an initial guess of each image in the training, validating and testing datasets using the approximant inverse model matrix, and then used as the input of BCNN for further training and prediction.

In order to efficiently compute the pseudoinverse of the large forward model matrix, ${{{{{\bf{H}}}}}}$, a computational approach was employed. The equation, ${{{{{\bf{H}}}}}}{{{{{{\bf{H}}}}}}}_{{{{{{\rm{inv}}}}}}}={{{{{\bf{I}}}}}}$ was solved one column at a time, where ${{{{{{\bf{H}}}}}}}_{{{{{{\rm{inv}}}}}}}$ is the pseudoinverse of ${{{{{\bf{H}}}}}}$ and ${{{{{\bf{I}}}}}}$ is the identity matrix. Thus, to calculate the ${i}^{{th}}$ column of ${{{{{{\bf{H}}}}}}}_{{{{{{\rm{inv}}}}}}}$, the LSQR method in Matlab was applied using ${{{{{\bf{H}}}}}}$ and the ${i}^{{th}}$ column of ${{{{{\bf{I}}}}}}$^35^. In the case of the simulations, which relied on the Russian Doll Hadamard matrix, the result was equivalent to the transpose of ${{{{{\bf{H}}}}}}$.

For the BCNN trained with the MNIST database, the Adam optimizer was used with a linearly decreasing learning rate starting from $5\times {10}^{-4}$ and ending with $5\times {10}^{-6}$. The batch size was chosen to be 40 and the BCNN was trained for 500 epochs to guarantee a complete training. The overall training time was approximately 7 minutes. For the BCNN trained with the STL-10 database, the Adam optimizer was used with a constant learning rate of $5\times {10}^{-4}$. The batch size was chosen to be 50 and the BCNN was trained for 200 epochs to guarantee a complete training. The overall training time was approximately 70 min.

### Experimental data acquisition and pre-processing

**Fig. 7:**
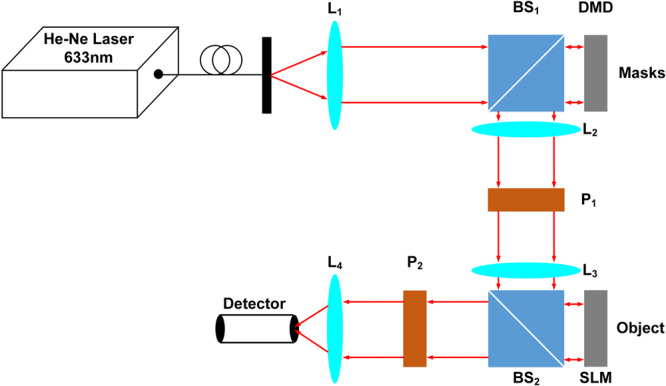
The imaging system. L_1_, L_2_, L_3_ and L_4_ are optical lenses whose focal lengths are 80 mm, 80 mm, 80 mm and 40 mm respectively. P_1_ is a horizontally polarized linear polarizer, and P_2_ is a vertically polarized linear polarizer. BS_1_ and BS_2_ are beam splitters. DMD is a digital micro-mirror device to display mask patterns. SLM is a spatial light modulator to display the object patterns. An sCMOS camera is used as the bucket detector by integrating all the pixels of each acquired image to produce the single-pixel signal.

Random grayscale illumination patterns were used in the experimental SPI. The images were taken from MNIST database^36^, normalized, converted to binary images and resized from 28 × 28 to 32 × 32 pixels. 1024 random grayscale illumination patterns each with a size of 32 × 32 were prepared as the full measurement basis. Then, the first 64 or 16 illumination patterns in the full basis were used to illuminate the objects, corresponding to a 16× or 64× compression ratio, respectively. Therefore, the corresponding 1D raw measurement data have a size of 64 × 1 or 16 × 1. The imaging system is shown in Fig. 7. A spatial light modulator (Pluto-Vis, Holoeye Photonics AG) is used to display each image and then the image is illuminated by a set of random grayscale sampling patterns which are displayed on a digital micromirror device^26^. The 1D measurement data were collected by a bucket detector. An sCMOS camera (Zyla 4.2 PLUS sCMOS, Andor Technology Ltd) was used as the bucket detector by integrating all the pixels of each acquired image to produce the single-pixel signal^26^. In the pre-processing step, an initial guess of each image in the dataset is reconstructed using the LSQR-approximated inverse model matrix, and then used as the input of BCNN for further training and prediction. The BCNN was trained on an experimentally acquired dataset of 800 images and tested on 100 images with the Bernoulli-distributed likelihood function. The batch size was chosen to be 40 and the BCNN was trained for 500 epochs to guarantee a complete training. The overall training time was approximately 7 min.

### Supplementary information

Supplementary Information

## Data Availability

The data to implement the BCNN in simulated 16× SPI with MNIST dataset is available at https://github.com/FMILab/Single-Pixel-Imaging-with-Uncertainty-Approximation. Other generated and/or analyzed datasets that support the findings of this study are available from the corresponding author upon reasonable request.

## Code availability

The code to implement and analyze the BCNN is available at https://github.com/FMILab/Single-Pixel-Imaging-with-Uncertainty-Approximation.
