# Supplementary material for: Approximating the uncertainty of deep learning reconstruction predictions in single-pixel imaging
Source: Commun Eng. Author manuscript; Available in PMC 2024 Mar 8. (PMC10923550; doi:10.1038/s44172-023-00103-1)
Supplement: Supplementary Information [file NIHMS1922104-supplement-Supplementary_Information.pdf]

Supplementary Information

Approximating the Uncertainty of Deep Learning Reconstruction Predictions in Single-pixel Imaging

Supplementary Note 1

Effect of noise on the BCNN performance

Since noise is one of the major challenges in imaging inverse problems, we sought to explore the effects of noise to both the image and uncertainty predictions in BCNN. The data simulation is the same as that with the MNIST database in the previous simulations except that varying SNR (defined in Eq. 22 in **Method**) levels of white Gaussian noise (SNR = 0dB, 5dB, 10dB, 15dB, 20dB or 25dB) were added to the 1D measurement data at the 16× compression ratio. The BCNN predictions are shown in Supplementary Fig. 1(a). The input images calculated from the LSQR-approximated inverse model matrix suffer at higher noise levels, while the predicted images from BCNN are more robust to noise. Besides, the predicted uncertainty generally matches well with the true absolute error at varying SNR levels. The regions where the predicted image from BCNN has larger errors are generally marked with higher uncertainty values in the predicted uncertainty. Similar to the findings in the section ***The BCNN predictions in the simulated SPI trained with the MNIST database*** in the main text, it can still be observed from the true absolute error and predicted uncertainty that most of the higher inaccuracies come from the edges of the image features at varying noise levels.

To quantitatively compare the performance, the mean and standard deviation of the MAE and SSIM for all the predicted images, and the correlation coefficients R between the true absolute error and the predicted uncertainty in the testing dataset at each noise level were calculated as shown in Supplementary Fig. 1(b). It shows that the performance of BCNN decreases as the SNR decreases from 25dB to 0dB. However, the fidelity of the corresponding result is still at a high level even if the data SNR is as low as 0dB, suggesting good robustness to noise. In addition, it is observed that the data uncertainty is dominant over the model uncertainty at higher noise levels, which can be shown quantitatively in Supplementary Fig. 1(c). This is reasonable since higher noise levels lead to higher variabilities in the data.

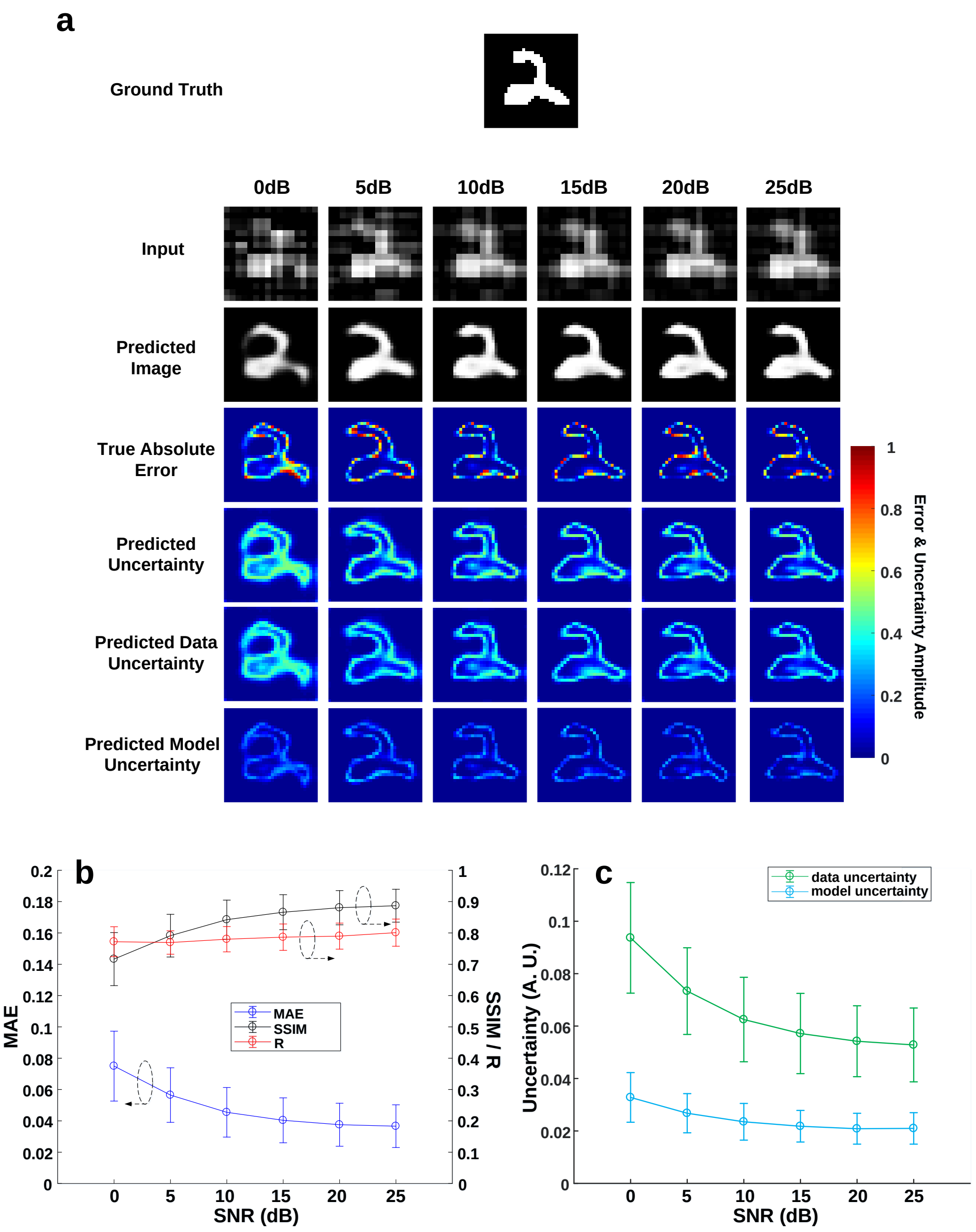

Supplementary Fig. 1 Effect of noise on the BCNN performance. (a) The BCNN results for a representative image in the testing dataset with varying SNR of noise added to the raw measurement data; (b) MAE, SSIM and R of the BCNN results with varying SNR of noise added to the raw measurement data; (c) Averaged pixel values of the predicted data and model uncertainties in the testing data with varying SNR. The error bars represent the standard deviation of the corresponding parameters from 100 testing images.

Supplementary Note 2

Training strategies

In this section, we sought to explore a different training strategy where the BCNN in the simulated SPI at the 8× compression ratio was trained with a mixture of 10,000 images from the MNIST database and 10,000 images from the STL-10 database at one time (Hybrid Training) and quantitatively compared its performance with the one trained on the same images from the two databases separately (Separate Training). In Separate Training, MAE, SSIM and the correlation coefficients R were calculated as the averaged MAE, SSIM and R of the predictions from the two databases. Hybrid Training has the same training and testing dataset as Separate Training where half of the dataset were from MNIST database and the other half were from STL-10 database, and the Laplacian-distributed (Hybrid Training (Laplacian)) and Bernoulli-distributed (Hybrid Training (Bernoulli)) likelihood functions were used separately for training. Supplementary Fig. 2 shows the quantitative comparisons in terms of MAE, SSIM and correlation coefficients R. For image predictions in terms of MAE and SSIM, Separate Training and Hybrid Training (Bernoulli) have similar performance, and both perform better than Hybrid Training (Laplacian). For uncertainty predictions in terms of correlation coefficients R, Separate Training and Hybrid Training (Laplacian) have similar performance, and both perform better than Hybrid Training (Bernoulli). The reason Hybrid Training (Bernoulli) has similar performance in image predictions as Separate Training is that the Bernoulli-distributed likelihood function fits the MNIST database and can also perform well in image predictions in the STL-10 database as we discussed in the prior section. However, the Bernoulli-distributed likelihood function cannot perform well in uncertainty predictions in STL-10 database as we discussed in the prior section, resulting in overall bad performance in uncertainty predictions. The reason Hybrid Training (Laplacian) has similar performance in uncertainty predictions as Separate Training is that the binary images in the MNIST database can be regarded as extreme images in the STL-10 database where the pixel values only distribute at the lower or upper bound. Therefore, Laplacian-distributed likelihood function can perform well in uncertainty predictions in Hybrid Training. However, these extreme images from MNIST database are harder to predict, resulting in the overall bad performance in image predictions in Hybrid Training (Laplacian).

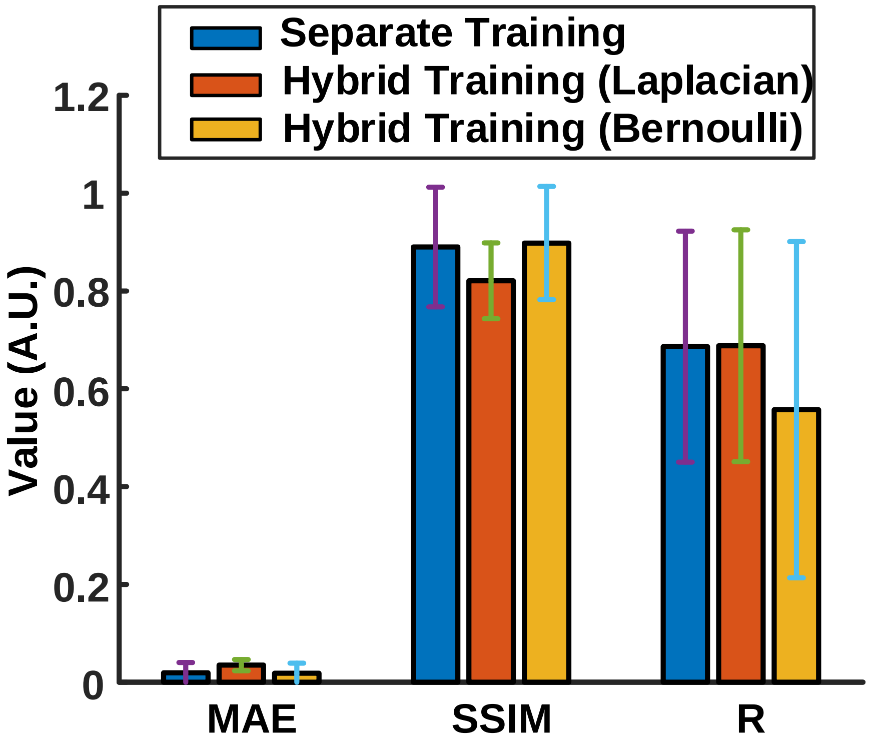

Supplementary Fig. 2 Quantitative (MAE, SSIM and R) comparisons of the BCNN performance among three training strategies in the simulated SPI at the 8× compression ratio trained with the MNIST and STL-10 database. The error bars represent the standard deviation of the corresponding parameters from 4000 testing images.

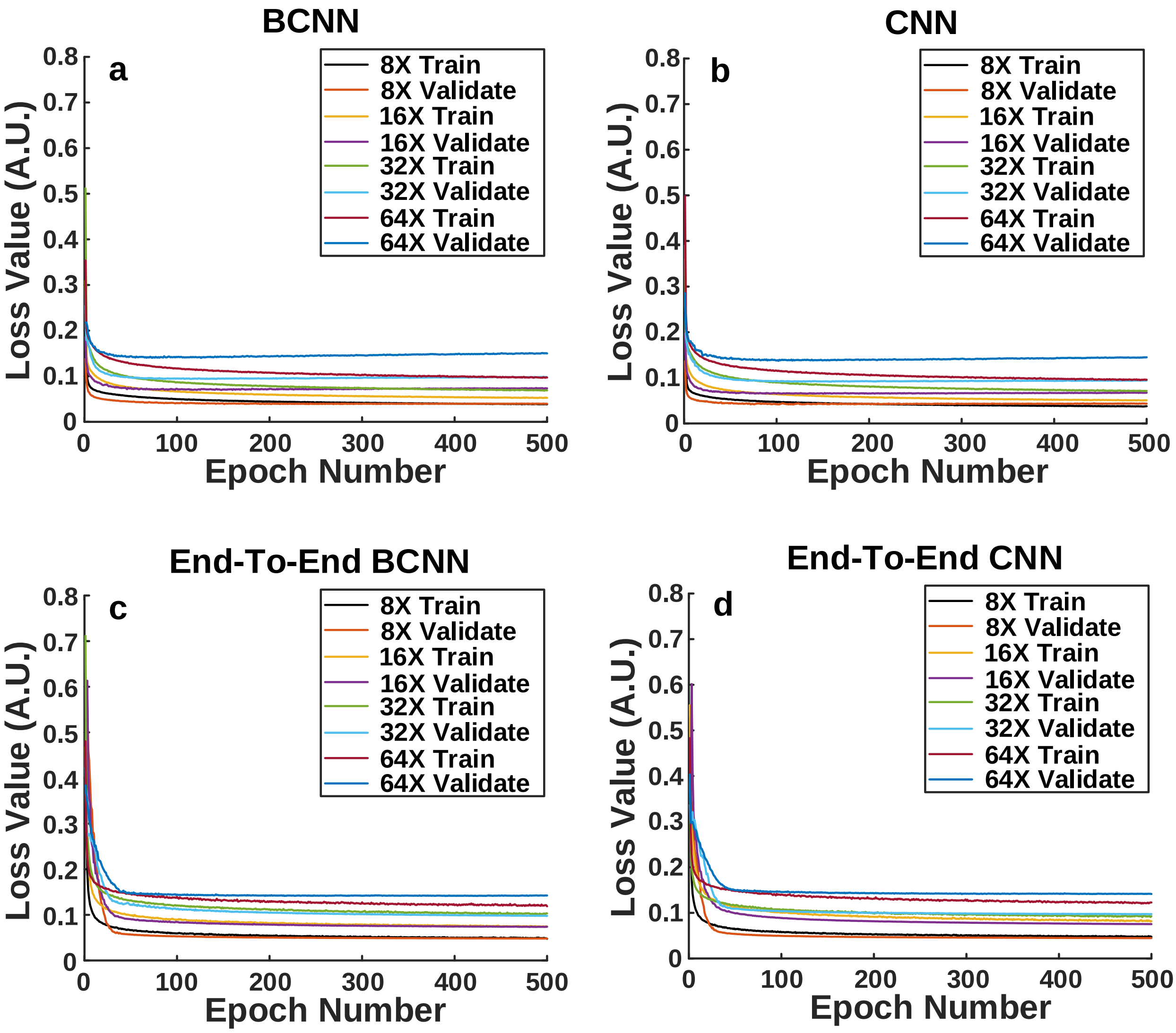

Supplementary Fig. 3 Training and validation curves in the simulated SPI trained with the MNIST database. (a) Training and validation curves of BCNN at 8×, 16×, 32× and 64× compression ratios. (b) Training and validation curves of CNN at 8×, 16×, 32× and 64× compression ratios. (c) Training and validation curves of End-To-End BCNN at 8×, 16×, 32× and 64× compression ratios. (d) Training and validation curves of End-To-End CNN at 8×, 16×, 32× and 64× compression ratios.

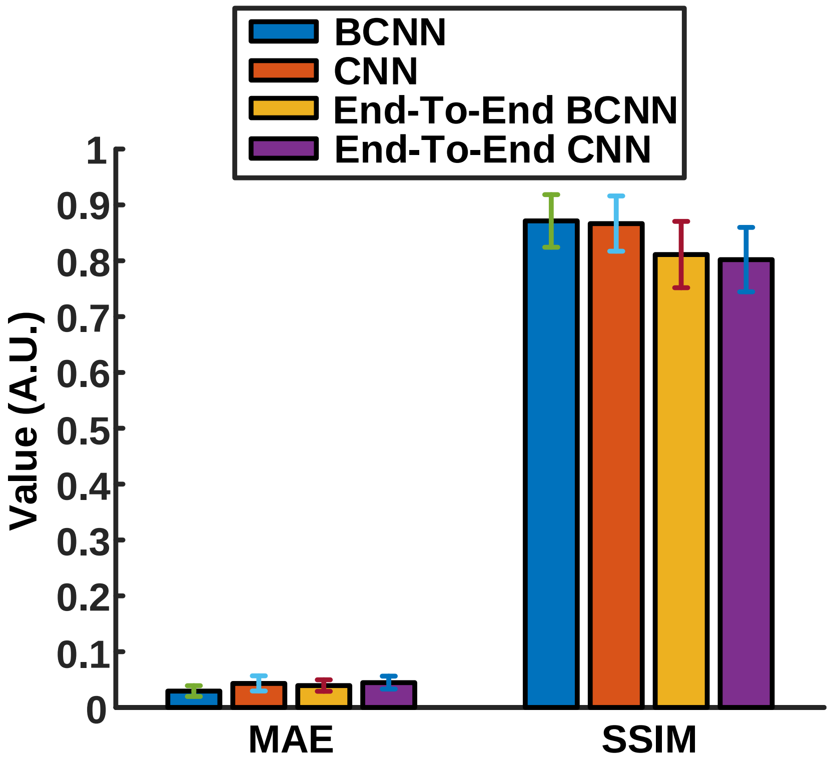

Supplementary Fig. 4 Quantitative (MAE and SSIM) comparisons among BCNN, CNN, End-To-End BCNN and CNN trained with the STL-10 database in the simulated SPI at the 4× compression ratio. The error bars represent the standard deviation of the corresponding parameters from 2000 testing images.

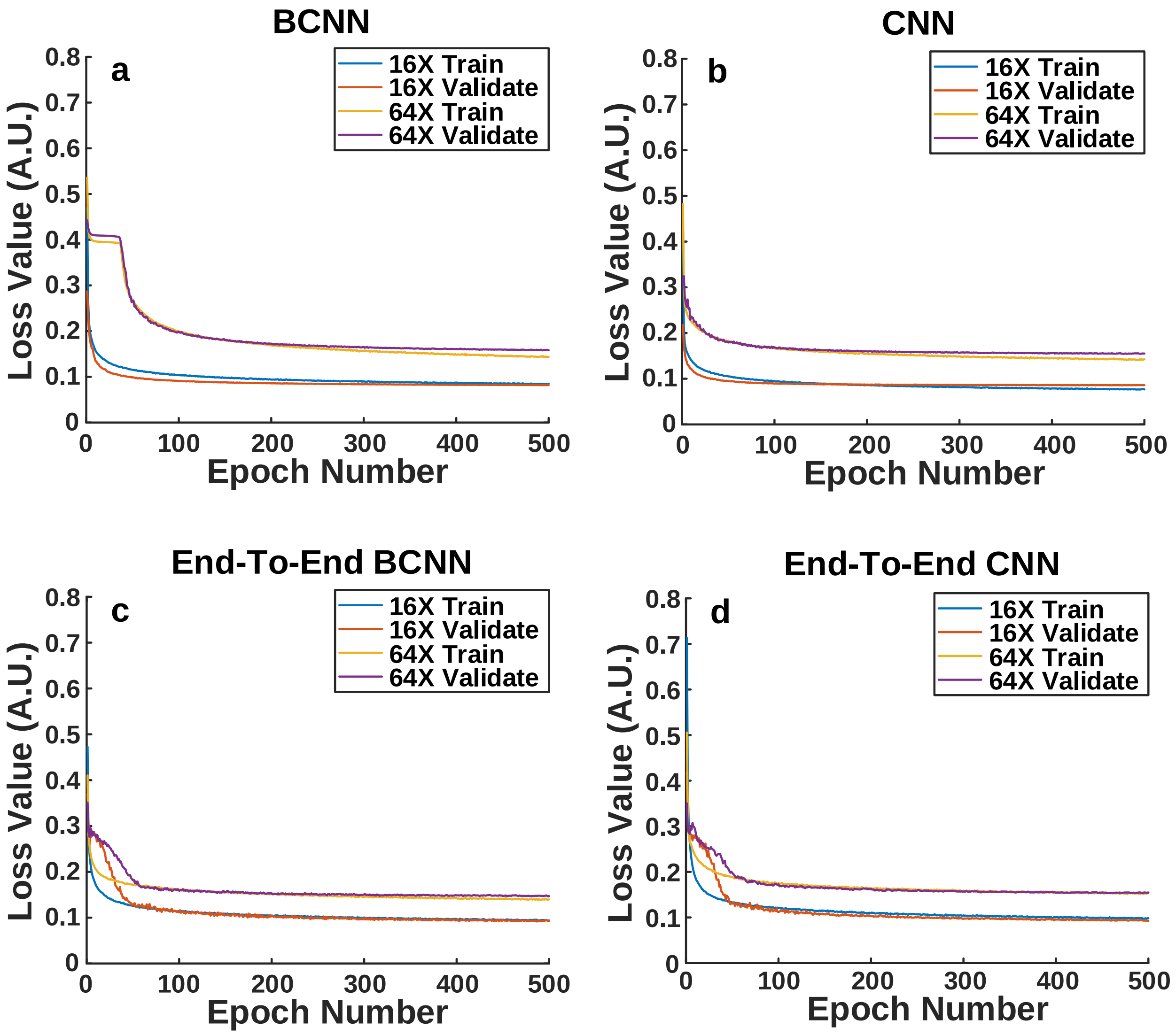

Supplementary Fig. 5 Training and validation curves in the experimental SPI trained with the MNIST database. (a) Training and validation curves of BCNN at 16× and 64× compression ratios. (b) Training and validation curves of CNN at 16× and 64× compression ratios. (c) Training and validation curves of End-To-End BCNN at 16× and 64× compression ratios. (d) Training and validation curves of End-To-End CNN at 16× and 64× compression ratios.

Supplementary Table 1. Quantitative comparisons among BCNN predictions with Laplacian-distributed, Gaussian-distributed and Bernoulli-distributed likelihood functions in simulated SPI with STL-10 database.

		MAE	SSIM	R
2×	Laplacian	0.0214	0.9285	0.4887
	Gaussian	0.0228	0.9194	0.5136
	Bernoulli	0.0203	0.9334	0.2017
4×	Laplacian	0.0294	0.8714	0.4922
	Gaussian	0.0288	0.8741	0.5003
	Bernoulli	0.0273	0.8865	0.2317
8×	Laplacian	0.0385	0.7801	0.4656
	Gaussian	0.0391	0.7756	0.4676
	Bernoulli	0.0366	0.7989	0.2379
16×	Laplacian	0.0505	0.6734	0.4515
	Gaussian	0.0502	0.6719	0.4608
	Bernoulli	0.0494	0.6797	0.2443
